# Frequency of HER2 Expression, MMR Deficiency, and PI3KCA Mutation in Pretreated Surgical Specimens of Patients with Esophageal Squamous Cell Carcinoma in Iran Cancer Institute

**DOI:** 10.30699/IJP.2023.563358.2989

**Published:** 2023-03-23

**Authors:** Samaneh Salarvand, Farzaneh Bagheri, Mahsa Gholizadeh, Sima Sharifi, Pooneh Panahi, Ebrahim Esmati, Marzieh Lashkari, Amirmohsen Jalaeefar, Mohammad Shirkhoda, Reza Shahsiah, Reza Ghalehtaki

**Affiliations:** 1 *Department of Clinical and Anatomical Pathology, Cancer Institute, Tehran University of Medical Sciences, Tehran, Iran*; 2 *Department of Radiation Oncology, Cancer Institute, Tehran University of Medical Sciences, Tehran, Iran *; 3 *Radiation Oncology Research Center, Cancer Research Institute, Tehran University of Medical Sciences, Tehran, Iran*; 4 *Department of Oncosurgery, Cancer Institute, Tehran University of Medical Sciences, Tehran, Iran*

**Keywords:** DNA Mismatch Repair, Esophageal Neoplasms, HER Family Receptor, Kinase, Squamous Cell Carcinoma

## Abstract

**Background & Objective:**

Iran is located in the esophageal cancer geographical belt. As multiple genetic alterations are responsible for the molecular pathogenesis of esophageal squamous cell cancer (ESCC), the role and frequency of *HER2 *expression, *MMR *deficiency, and *PI3KCA* mutation are not well defined.

**Methods:**

We carried out *HER2/neu* expression, *dMMR/MSI* high, and *PI3KCA* mutation analysis in specimens of patients with ESCC. We accessed archival tissue blocks related to specimens of 68 ESCC cases at the time of surgery following neoadjuvant chemoradiation. These patients underwent surgery during 2013-2018 at the Cancer Institute of Iran affiliated with the Tehran University of Medical Sciences in Tehran.

**Results:**

None of the patients showed *HER2* expression, *dMMR/MSI* high, or *PI3K* mutations.

**Conclusion:**

*dMMR/MSI-H* and *PI3KCA* mutation and *HER2 *expression may not be reliable andfrequent targets for systemic therapy in patients with esophageal SCC.

## Introduction

Cancer, as a chronic, costly, and life-threatening disease, is one of the most critical health challenges in the world. Esophageal cancer is considered the eighth most common cancer and the sixth most frequent cause of death worldwide. In 2018, according to GLOBOCAN data, the global rate of esophageal squamous cell carcinoma (ESCC) incidence and death were 572,000 and 509,000, respectively (1). As a multifactorial disease, the development of gastric and esophageal cancers is influenced by both genetic and environmental factors. Therefore, its incidence is expected to vary throughout the world. The reports have indicated that the disease is relatively common in Iran. In a report by World Health Organization, esophageal cancer is the ninth most common malignancy in Iran (2). What is often referred to as the “esophageal cancer belt” originates from northern Iran and passes through Central Asia to reach northern central China (3). Reports from the Cancer Registry of the International Agency for Research on Cancer indicated that the prevalence of esophageal cancer in northern Iran was 165 per 100,000 male individuals and 195 per 100,000 female individuals (2). Squamous-cell carcinoma (SCC) is the predominant subtype of esophageal cancer in Iran (4). 

The overall 5-year relative survival of esophageal cancer between 2012-2018 in SEER geographic areas is 20.6% (5). Systemic therapy has limited effectiveness in metastatic esophageal cancer, with responses observed in only 20-40% of the population, and a median survival time of around 9 months (6). Based on the literature, 5-year survival in ESCC is low, and cancer metastases, rather than primary cancer, are the main cause of as high as 90% of cancer-related deaths (7). Metastases are the final step of a cellular biological process termed the invasion-metastasis cascade. This process involves enhanced cell mobility and intravasation, transit in lymphatic and blood vasculature, extravasation, and finally growth at a new location (7, 8). The identification of the biological process and genetic pathways that cause invasion and metastasis will facilitate the development of new treatment modalities. This study aimed to evaluate some possible pathways and mutations that contribute to the development of invasion and metastasis of ESCC.

Latest advances in gene profiling techniques and the development of metastatic cell models have been crucial in the identification of genes and pathways associated with tumor metastases (9). Therefore, to improve the risk-adapted therapeutic strategies and stratify patients to enroll in future clinical trials on actionable molecular pathways in ESCC, we need the identification of new prognostic or predictive molecular markers. The reports indicated targets to treat malignancies, such as breast, stomach, and esophagus (10, 11).

The epidermal growth factor receptor family comprises four homologous molecules, including *EGFR, c-erbB-2, cerbB-3, *and* c-erbB-4*. A study suggested that the abnormal activation of kinase activity in these receptors has a pivotal role in the development and progression of ESCC (12). The tyrosine kinase function of these receptors is essential for intracellular signaling and cell transformation in the proliferation, differentiation, and maturation of the embryonic intestine (13, 14). Although the detection of gene amplification might be a mainstay factor in decision-making for treatment, data are scarce regarding *HER2*/*neu* amplification in patients with ESCC.

Phosphatidylinositol 3-kinases (PI3K) are a ubiquitous lipid kinase family that catalyzes the phosphorylation of molecules phosphatidylinositol (PI), PI (4) P, and PI (4, 5) P2 into PI (3) P, PI (3, 4) P2, and PI (3, 4, 5) P3, respectively (15). These products of lipid entity can then activate a variety of downstream events that regulate a wide spectrum of essential cellular processes, including growth and cell cycle progression, apoptosis and migration, metabolism, and vesicular trafficking. This overactivation might result in cell function dysregulation by interrupting apoptosis and inducing proliferation, eventually triggering tumor formation (16).

Microsatellite instability (MSI) is identified by extensive somatic alterations in the nucleotide repeat sequences (microsatellites), which is almost always a reflection of a germline mutation in any of the mismatch repair* (MMR*) genes, mostly *MLH1* and *MSH2* (17). In colorectal cancer, MSI is associated with clinicopathological characteristics, such as proximal location and poor differentiation, low metastasis potential, and eventually a better prognosis (18). Based on the previous data about these targets in the treatment of patients with ESCC, the present study attempted to assess the status of *MSI *phenotype and *HER2* expression and mutation in the *PI3K* pathway in ESCC in Iranian patients.

## Material and Methods

**Study Design**

This retrospective study was carried out on the esophagectomy samples obtained from patients with ESCC at the time of surgery. The patients attended the Cancer Institute of Iran affiliated with the Tehran University of Medical Sciences in Tehran, Iran, during 2013-2018.

**Inclusion and Exclusion Criteria**

In this study, patients without gender and age restrictions were evaluated. We included specimens with SCC histology in surgical specimens of patients after neoadjuvant chemoradiotherapy. Those with complete response to preoperative treatment were kept out due to a lack of any malignant tissue to perform adequate testing.

**ESCC Cases and Specimens**

First, the records of patients with esophageal cancer extracted from the archives of the Radiation Oncology Department of Imam Khomeini hospital in Tehran, information about the stage of the disease, grade and survival rate, neoadjuvant treatment of tumor, location based on distance from incisors according to endoscopy or endoscopic ultrasound or computed tomography scan, and other characteristics were registered in the designated data forms. The TNM Classification of Malignant Tumors staging was determined according to the surgical pathology reports. In this study, we utilized the archived formalin-fixed paraffin-embedded blocks from the tumor tissues of 68 ESCC patients at the time of surgery. 

The used samples included the tissues prepared during surgery from patients with esophageal cancer. The standard and routine method used in the pathology section was employed to prepare the tissue, which briefly includes several steps, namely the preparation of appropriate initial tissue incision, fixation, and processing with a tissue processor, performing appropriate incisions using a microtome, and preparing slides, dying slides, and assembling them. The tumor site on the slide was marked under a microscope and matched with the paraffin block.

Most of the cases received neoadjuvant chemoradiation and chemotherapy. In this setting, only those with incomplete responses to neoadjuvant therapy could be evaluated because there was no remnant tumor in cases with complete response, and there was no access to the preneoadjuvant biopsy samples. Gender, primary-tumor site, histological grade and the extent of normal tissue infiltration, lymphatic and venous invasion, and surgical margin and pathological stage, tumor regression grade (TRG), and type of neoadjuvant therapy were reviewed for all cases. 

**Laboratory Techniques**

Immunohistochemistry (IHC) was used to detect *HER2*/*neu* amplification. The IHC reactions were carried out by the technique named streptavidin-biotin-peroxidase complex (StreptABC, DAKO, Denmark). The sections from tissues were then de-paraffinized and incubated in a pressure cooker containing citrate buffer for retrieval of antigens. In the next step, the activity of endogenous peroxidase was inhibited by a 3% hydrogen peroxide solvent. The sections were then exposed to polyclonal primary antibodies against *HER2/neu* (1:500, A0485, DAKO, Denmark). In the following step, the sections were incubated in a secondary biotinylated antibody of an LSAB+ peroxidase Kit (DAKO, K0690, Denmark), and then incubated with streptavidin HRP (DAKO, Denmark), and were counterstained using hematoxylin dye. Immunohistochemical analyses of *HER2 *expression describe the positivity of tumor cells and their staining pattern.

Deoxyribonucleic acid (DNA) isolation and polymerase chain reaction (PCR) reactions were required to detect the presence of mutations in *PI3K*. The paraffin block was selected for two cuts from the marked tumor area using a scalpel and inserted into a microtube for each tissue sample. New blades were used to cut each tissue to prevent the carry-over effect. QIAGEN Gene Read DNA FFPE Kit (QIAGEN, Germany) was utilized to extract DNA based on the instructions. After extraction, DNA quality was evaluated by absorbance ratios at 260/280 nm. Table 1 shows primer sets and amplification programs. Amplification was performed on a LightCycler 96 System (Roche, Basel, Switzerland). 

**Fig. 1:**
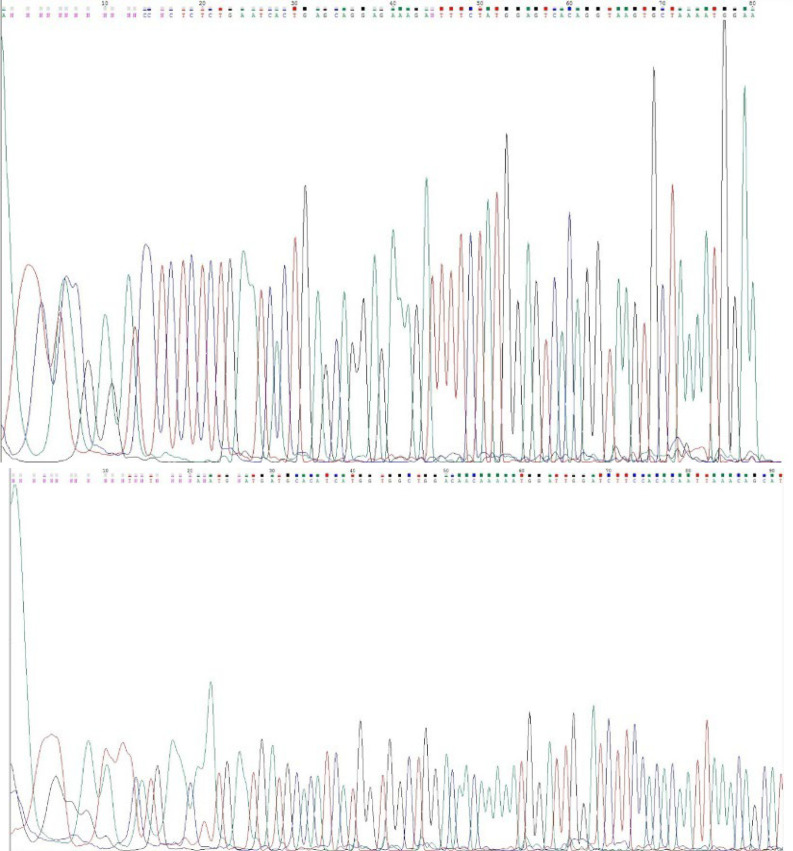
Sequences of exon 9 (A) and exon 20 (B) of PIK3A

The PCR products were sent to Kowsar Biotech Company (KBC®, Tehran, Iran) to sequence amplified gene fragments using Sanger sequencing. The results included electropherogram files provided by the company to the researcher. Finally, the presence of a gene mutation was determined using gene alignment on the peak scanner software and comparing the obtained sequence with the standard *PIK3CA* sequence.

In this study, there were three hotspot mutations in the *PIK3CA* gene, including *E542K* and *E545K* in exon 9 and *H1047R* in exon 20 by Sanger sequencing after PCR amplification. As shown in Figure 1, the sequences of *GAA* at 1624, *GAG* at 1634 in exon 9, and *CAT* at 3139 and 3140 in exon 20 were wild-type (normal), and no point mutation was recorded. 

DNA isolation and PCR reaction were required to assess the *MSI* status. QIAGEN Read TM DNA FFPE Kit was used to extract tissue DNA, and the extraction process was performed according to the kit instructions. After extraction, DNA quality was evaluated by absorbance ratios at 260/280 nm. 

For performing the PCR reaction, suitable primers were used to amplify the number of copies of five human DNA microsatellite sequences based on the Bethesda panel of five microsatellite markers. Forward and reverse primers for detection of the amplifying sequences *NR27, BAT26, BAT25*,* NR24*, and *NR21* were requested from Macrogen (Geum Chun-Gu, Seoul, Korea) with their already defined sequences (19). The PCR reaction was performed in a LightCycler Nanomachine (Roche Diagnostics GmbH, Mannheim, Germany).

The melting graphs were evaluated, and the samples were delivered for capillary electrophoresis if acceptable. Electrophoresis results were analyzed by peak scanner software (version 1.0; Applied Biosystems, CA, USA). Detected alterations in two or more markers were considered *MSI-H*, while gene mutations in only one marker were considered as *MSI-L*.

**Table 1:** PCR primer sets and amplification programs

Target gene		Forward primer	Reverse primer
*PI3KCA*	*Exon 9*	5’- GGG AAA ATG ACA AAG AAC AGC TC-3’	5’- TCC ATT TTA GCA CTT ACC TGT GAC-3’
	*Exon 20*	5’- CTA GCC TTA GAT AAA ACT GAG CAA G-3’	5’- AGA GTT ATT AAC AGT GCA GTG TGG A-3’
	PCR protocol: 50 cycles, 95˚C for 15 minutes, 95˚C for 20 seconds, 60˚C for 20 seconds, 72˚C for 20 seconds, and 72˚C for 5 minutes		

**Statistical Analysis**

There was no power analysis for this study, and all accessible specimens underwent laboratory testing to detect desired mutations or expressions. The quantitative variables were presented using central tendency parameters. The qualitative variables were expressed using frequency and proportion. Median survival values in terms of months were expressed using the Kaplan-Meier method. SPSS software version 20 (SPSS Inc., Chicago, Ill., USA) was used for statistical tests**.**

## Results

This study examined 68 patients with ESSC and one patient with gastroesophageal junction SCC. Table 2 shows the characteristics of the patients. Almost all patients underwent esophagectomy. Two patients did not undergo esophagectomy, including one with partial gastrectomy and one who was biopsied with suspicion of interval metastases. According to the operative reports, 17 (25%) and 49 (72%) patients had intermediate (ypT1-2 or N0) and poor (ypT3-4 or N+) pathologic responses to the preoperative treatment, respectively. In other words, based on the modified Ryan system, among those who underwent tumor resection, there were 5 (7.6%), 34 (51.2%), and 27 (40.9%) patients with TRG 1, 2, and 3, respectively. Moreover, 5 (7.4%) patients had local recurrence, and 36 (52.9%) patients had distant metastases. The liver was the most common site for distant metastases in 18 (26.5%) subjects.

The median follow-up of the surviving patients was 57 months using the reverse Kaplan-Meier method. The median overall and disease-free survival were 25 and 18 months, respectively. Accordingly, the 5-year rates of overall survival and disease-free survival were 24% and 17%, respectively. In this study, 68 samples were undergone to IHC evaluation, and none were observed to be *HER2* overexpressed. This study examined 39 out of 68 patients for *PI3K* mutation and *MSI* status. None of the patients had high *dMMR*/*MSI* or *PI3K* mutations. 

**Table 2:** Baseline, clinical and surgical characteristics of the patients

Characteristic		Number	Percentage
Gender	Male	34	50%
	Female	34	50%
Tumor location	Proximal esophagus	3	4.4%
	Middle esophagus	22	32.4%
	Distal esophagus	42	61.8%
	Missing	1	1.4%
Depth of invasion	T1	2	2.9%
	T2	19	27.9%
	T3	42	61.8%
	T4	3	4.4%
	Missing	2	2.9%
Differentiation	Well	13	19.2%
	Moderately	34	50%
	Poorly	20	29.4%
	Missing	1	1.4%
Lymphovascular invasion	Yes	40	58.8%
	No	27	39.8%
	Missing	1	1.4%
Lymph node metastases	0	40	58.8%
	1	17	25%
	2	6	8.8%
	X	4	6%
	Missing	1	1.4%
Surgical margin	Positive	15	22.1%
	Negative	51	75%
	Missing	2	2.9%
Surgical stage	IB	5	7.4%
	IIA	5	7.4%
	IIB	17	25%
	IIIA	5	7.4%
	IIIB	5	7.4%
	IIIC	2	2.9%
	Missing	29	42.6%
Perineural invasion	Absent	30	44.2%
	Present	37	54.4%
	Missing	1	1.4%
Tumor regression grade	TRG1	5	7.6%
	TRG2	34	51.2%
	TRG3	27	40.9%
	Missing	2	2.9%
Type of neoadjuvant therapy	None	1	1.4%
	RT+CHT	64	94.2%
	CHT without RT	3	4.4%

## Discussion

At the global level, the mortality of esophageal cancer is projected to increase among male and female subjects, more evident in less developed countries (20). There is wide geographical variation in the incidence of ESCC throughout the world. This fact might indicate that ESCC has a heterogenous and diverse pattern in its molecular and clinical manifestations (21). Various molecular pathways lead to cancer in various populations. The evaluation of mutations in disease-related genes is one of the practical tools for identifying diseases and predicting their probability of occurrence. Additionally, to have an opportunity for individualized treatment, identifying molecular characteristics of the patients’ tumor is of utmost importance to refine outcomes and keep down the exposure to unnecessary toxicities.

Some known oncogenic pathways can be observed by referring to the Kyoto Encyclopedia of Genes and Genomes pathway database (22). However, this database has not registered esophageal cancer yet, indicating the importance of further research on the molecular status of esophageal cancer.

The analysis of IHC studies showed that 8.6% of the cases were positive for the expression of *HER2* (23). In a few studies that have so far reported the frequency of *HER2* expression in ESCC, there is a considerable discrepancy between 0% and 64% (20-22). This variability might have resulted from differences in the patient selection based on ethnicity and genetic background, preoperative treatment status or stage of presentation, the variations in opted IHC techniques or antibody sources, or even various criteria for evaluating the expression. This study used IHC to detect *HER2* expression; however, the detection of *HER2* gene amplification using fluorescence in situ hybridization might increase accuracy and make useing of anti-*HER2* targeted therapies more beneficial.

The frequency and the clinical relevance of *MSI* in ESCC are not well recognized. There are discrete and even contradictory MSI studies in the ESCC population. The reported rate of *MSI *in ESCC largely differs among the previous investigations from 0% to 40% (24). However, relatively high levels of *MSI-L* status were seen in ESCC, compared to those of colorectal or gastric cancers (25). The* MSI* is considered a predictive marker of tumor response to immunotherapy and can be essential to examine the status of this genetic instability mechanism in metastatic ESCC. In this study, there were no cases of *MSI*. Two standard recommended methods are there to detect tumor* MMR* deficiency, namely PCR and loss of *MMR* protein expression by IHC (26). The PCR methods and criteria used to determine the *MSI* phenotype are pentaplex and HT17 repeat. A 17 mononucleotide repeat of HSP110 (HT17) is critical for correct splicing of this chaperone and improving current standard molecular methods to detect *MSI* in colorectal cancer (27). The pentaplex PCR detection of *MMR* protein expression was used in this study. 

The* PIK3CA* mutation has been reported with various frequencies, and its association with prognosis has not been consistent in available studies. In ESCC, although *PIK3CA* mutation has been detected in 2% to 12% of studied cases, its prognostic or predictive role is still unknown (10). Several reasons might explain the difference between the observations of the present study and other studies. First, in the current study, the Sanger sequencing method was used for the mutational analysis of *PIK3CA*. A comparison study by Arsenic et al. showed that next-generation sequencing has superior sensitivity in detecting* PIK3CA* mutation over the Sanger sequencing technique (28).

In addition to the above-mentioned issues, lack of access to the tissue samples before neoadjuvant therapy (due to the initial referral of patients to other medical centers) led to the exclusion of samples with a complete response to preop treatment from the study that roughly comprised 30% of all ESCC patients. However, the omission of these samples could not be a causative factor for such low rates of *HER2* overexpression,* PI3K* mutation, and *MSI-H* status in the present study, compared to those of other studies. The present data indicated that none of the patients with ESCC had *HER2* amplification and/or *MSI-H status* or *PI3KCA* mutation in Iran; nevertheless, this topic in ESCC requires further investigation. 

## Conclusion

The results of the current study indicated that *dMMR/MSI-H* and *PI3KCA* mutation and *HER2* expression are unreliable, frequent targets for systemic therapy in patients with ESCC. Other investigations are encouraged to detect other applicable mutations.

## Author contributions

Reza Ghalehtaki: Designed the analysis, collected data, contributed data or analysis tools, performed the analysis, and wrote the paper. Samaneh Salarvand: received and designed the analysis, collected data, and contributed data or analysis tools. Mahsa Gholizadeh: Collected data, and contributed data or analysis tools. Farzaneh Bagheri: Wrote the paper. Sima Sharifi: Collected data, contributed data or analysis tools, Pooneh Panahi: Collected data, contributed data or analysis tools. Ebrahim Esmati: Wrote the paper. Marzieh Lashkari: Collected data and wrote the paper. Reza Shahsiah: Received and designed the analysis, collected data, and contributed data or analysis tools. Amirhosein Jalaeefar: Collected data and wrote the paper. Mohammad Shirkhoda: Collected data and wrote the paper. 

All authors read and approved the final manuscript. 

## Conflict of Interest

The authors declared no conflict of interest.

## Ethics Approval

This study was performed in accordance with the principles of the Declaration of Helsinki. Approval was granted by the Ethics Committee of Tehran University of Medical Sciences (IR.TUMS.VCR.REC.1397.020, IR.TUMS.IKHC.REC.1397.325)

## Data Availability

Data sharing is not applicable to this article as no datasets were generated or analyzed during the current study.

## References

[1] Bray FF, Ferlay J, Soerjomataram I, Siegel RL, Torre LA, Jemal AJ (2020). Erratum: Global cancer statistics 2018: GLOBOCAN estimates of incidence and mortality worldwide for 36 cancers in 185 countries. Ca Cancer J Clin.

[2] Khademi H, Kamangar F (2012). Esophageal cancer incidence trends in northeastern Iran: comparing rates over 36 years.

[3] Kmet J, Mahboubi E (1958). Mechanized Information Storage, Retrieval and Dissemination.

[4] Chen J, Kwong DL, Cao T, Hu Q, Zhang L, Ming X, Chen J, Fu L, Guan X (2015). Esophageal squamous cell carcinoma (ESCC): advance in genomics and molecular genetics. Dis Esophagus.

[5] Esophageal Cancer - Cancer Stat Facts.

[6] Sakaguchi M, Maebayashi T, Aizawa T, Ishibashi N, Saito T (2018). Clinical results of multimodality therapy for esophageal cancer with distant metastasis. J Thoracic Dis.

[7] Li B, Xu WW, King A (2017). Significance of PI3K/AKT Signaling Pathway in Metastasis of Esophageal Squamous Cell Carcinoma and Its Potential as a Target for Anti-Metastasis Therapy.

[8] Valastyan S, Weinberg RA (2011). Tumor metastasis: Molecular insights and evolving paradigms. Cell.

[9] Lin KT, Yeh YM, Chuang CM (2015). Glucocorticoids mediate induction of microRNA-708 to suppress ovarian cancer metastasis through targeting Rap1B. Nature Communications.

[10] Shigaki H, Baba Y, Watanabe M (2013). PIK3CA mutation is associated with a favorable prognosis among patients with curatively resected esophageal squamous cell carcinoma. Clin Can Res.

[11] Yokota T, Serizawa M, Hosokawa A PIK3CA mutation is a favorable prognostic factor in esophageal cancer: molecular profile by next-generation sequencing using surgically resected formalin-fixed, paraffin-embedded tissue.

[12] Andl CD, Mizushima T, Nakagawa H (2003). Epidermal growth factor receptor mediates increased cell proliferation, migration, and aggregation in esophageal keratinocytes in vitro and in vivo. J Biol Chem.

[13] Weaver LT, Walker WA (1988). Epidermal growth factor and the developing human gut. Gastroenterology.

[14] Wieduwilt MJ, Moasser MM The epidermal growth factor receptor family: Biology driving targeted therapeutics.

[15] Liu P, Cheng H, Roberts TM, Zhao JJ Targeting the phosphoinositide 3-kinase (PI3K) pathway in cancer.

[16] Yang J, Nie J, Ma X, Wei Y, Peng Y, Wei X (2019). Targeting PI3K in cancer: Mechanisms and advances in clinical trials. Mol Can.

[17] Cortes-Ciriano I, Lee S, Park WY, Kim TM, Park PJ (2017). A molecular portrait of microsatellite instability across multiple cancers. Nature Communications.

[18] Jung MK, Shin US, Ki YJ, Kim YB, Moon SM, Sung SJ (2017). Is the location of the tumor another prognostic factor for patients with colon cancer?. Annals of Coloproctology.

[19] Sorokin M, Rabushko E, Efimov V (2021). Experimental and Meta-Analytic Validation of RNA Sequencing Signatures for Predicting Status of Microsatellite Instability. Front Mol Biosci.

[20] Gupta B, Kumar N (2017). Worldwide incidence, mortality and time trends for cancer of the oesophagus. Eur J Cancer Prev.

[21] Abnet CC, Arnold M, Wei WQ Epidemiology of Esophageal Squamous Cell Carcinoma HHS Public.

[22] Kanehisa Laboratories. KEGG.

[23] Egebjerg K, Garbyal RS, Hasselby JP, Baeksgaard L, Mau-Sørensen M (2021). Prevalence of HER2 overexpression and amplification in squamous cell carcinoma of the esophagus: A systematic review and meta-analysis. Critical Reviews in Oncology/Hematology.

[24] Campanella NC, Lacerda CF, Berardinelli GN (2018). Presence of microsatellite instability in esophageal squamous cell carcinoma associated with chagasic megaesophagus. Biomark Med.

[25] Zhu M, Jin Z, Hubbard JM (2021). cancers Management of Non-Colorectal Digestive Cancers with Microsatellite Instability.

[26] Svrcek M, Lascols O, Cohen R ( 2019). Short Title: Standard for Screening and Diagnosis of MSI/MMR Deficient Tumor.

[27] Buhard O, Lagrange A, Guilloux A (2016). HSP110 T17 simplifies and improves the microsatellite instability testing in patients with colorectal cancer. J Med Gen.

[28] Arsenic R, Treue D, Lehmann A (2015). Comparison of targeted next-generation sequencing and Sanger sequencing for the detection of PIK3CA mutations in breast cancer. BMC Clin Pathol.

